# Multiple right-sided pulmonary nodules: metastatic cancer or resectable early stage tumor?

**DOI:** 10.1186/1749-8090-6-105

**Published:** 2011-09-05

**Authors:** Ugo Cioffi, Federico Raveglia, Matilde De Simone, Vincenzo Valenti, Michele M Ciulla, Alessandro Baisi

**Affiliations:** 1 Department of Surgery, University of Milan, Fondazione IRCCS Ospedale Maggiore Policlinico, Milan, Italy; 2 Thoracic Surgery Unit, Azienda Ospedaliera San Paolo, Milan, Italy; 3 Division of Pulmonary Medicine, IRCCS Policlinico San Donato, San Donato Milanese, Milan, Italy; 4 Department of Respiratory and Cardiovascular Disease, Centro di Fisiologia Clinica e Ipertensione, Laboratory of Clinical Informatics and Cardiovascular Imaging, University of Milan, Milan, Italy

**Keywords:** lung cancer, surgery, metastasis, PET/CT scan

## Abstract

The aim of this paper is to focus attention on complex cases of lung disease that may benefit from being managed outside formal guidelines. *A *52 year-old man who had previously undergone a laryngectomy for squamous cell carcinoma, presented with a 1.2 cm nodule in the right upper pulmonary lobe. Three months later a new CT scan found that the nodule had slightly increased in size and also detected two new smaller nodules in the middle lobe. A PET/CT scan showed metabolic hyperactivity of all nodules. Since needle aspiration of the upper one revealed malignant cells, the patient was considered to be suffering from metastatic cancer and started on chemotherapy. At follow-up both CT and PET scans found a significant reduction in volume and activity of the lower nodules but no change in the upper one. At diagnostic thoracoscopy, histology demonstrated that the upper nodule was an adenocarcinoma while the lower ones were inflammatory. An upper lobectomy and systematic nodal dissection were therefore performed. Histology established a diagnosis of upper pulmonary adenocarcinoma and sarcoidosis. Our report suggests that in complicated oncologic cases in which non-invasive diagnostic tools yield incongruous results surgery should be considered without delay.

## Introduction

We report the case of a patient with three right pulmonary nodules and a previous advanced metastatic carcinoma of the pharynx. Follow-up and treatment were managed by a team of surgeons and oncologists according to the American College of Chest Physicians (ACCP) evidence-based clinical practice guidelines [1]. The complexity of the case lies in its being open to different diagnostic interpretations and in the impossibility of obtaining sufficient clarification by means of non-invasive techniques. The non-conventional ultimate decision to perform a surgical biopsy of all nodules led to an unexpected diagnosis which entailed a better prognosis and a change in the therapeutic strategy. The aim of this paper is to focus attention on the role of timely surgery in the diagnosis of complicated oncologic cases that may at some point require a more personalised approach, outside formal guidelines.

## Case report

**Figure 1:**
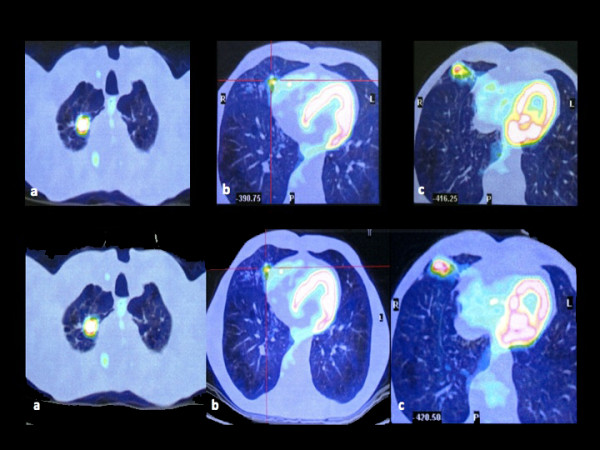
**Original PET/CT scan images obtained before (Upper Panel) and after (Lower Panel) chemotherapy showing the three nodules within the right upper (Panels a) and middle (Panels b,c) pulmonary lobes**. The nodule in the upper lobe shows a stable activity (SUV from 10.6 to 9 g/ml) whereas the nodules in the middle lobe show a significant decrease in activity (SUV from 5.1 to 2.6 g/ml) and an almost complete abolishment of activity (SUV from 2.9 to > 0.9 g/ml).

A 52 year-old male who was a heavy smoker presented with a single right upper lobe pulmonary nodule 1.2 cm in diameter. In May 2007 he had developed a tongue carcinoma which had been treated by a supraglottic laryngectomy extended to the tongue base, temporary tracheotomy and elective neck nodal dissection. Histology was pharyngeal squamous cell carcinoma, pT2N2M0G2 with minimal invasion of the deep tongue surgical edge. The advanced tumor stage had required adjuvant radiotherapy (65 Gy) and chemotherapy (two cycles of cisplatin). In September 2008 a CT scan revealed the 1.2 cm irregular nodule in the upper pulmonary lobe. The radiologic report stated it was suggestive of scarring tissue. Bronchoscopy and bronchoalveolar lavage cytology were negative. The fully informed patient chose a non-aggressive management approach. Therefore, according to the ACCP evidence-based clinical practice guidelines, a serial CT scan observation was adopted [2]. In December 2008 a chest CT scan showed a slight increase in size of the right upper lobe nodule (1.4 cm) and the appearance of two nodules in the middle lobe. The dimensional increase of the nodule and the occurrence of new lesions rendered unlikely the hypothesis of a benign disease and shifted the odds in favour of primary or metastatic lung cancer. A PET/CT scan was therefore performed on January 2009 [3] and it revealed a high FDG uptake of the right upper lobe nodule (Standard Uptake Value (SUV) max = 10.6 g/ml) and a significant FDG uptake of both the middle lobe nodules (SUV = 2.9 g/ml and 5.1 g/ml), a metabolic behaviour consistent with carcinoma according to the nuclear medicine report (Figure 1). Before starting any treatment the oncologists needed to obtain a tissue diagnosis. To this purpose a CT-guided trans-thoracic needle aspiration (TTNA) was deemed to be the easiest method, considering the position of the nodules [4]. Improperly, only one nodule underwent needle aspiration and it was targeted to the upper nodule because of its greater size, higher metabolic activity and peripheral location. Cytology demonstrated malignant epithelial cells and on these grounds several diagnoses were possible, such as multiple lung metastases of pharyngeal carcinoma, primitive lung carcinoma with pulmonary metastases or both primitive and secondary lung carcinoma. In any case, the diagnosis of advanced cancer was supported by the metabolic hyperactivity of all nodules.

The hypothesis of multiple lung metastases of pharyngeal carcinoma seemed the most likely and the patient underwent chemotherapy with cisplatin and vinorelbine. After three chemotherapy cycles a CT scan was repeated. It showed that the right upper lobe nodule was unchanged and that the two middle lobe nodules were significantly reduced. A PET/CT scan confirmed this pattern of evolution, showing that the activity of the upper lobe nodule was stable (SUV = 9 g/ml versus 10.6 g/ml) whereas the activity of the middle lobe nodules was in one case significantly reduced (SUV = 2.6 g/ml versus 5.1 g/ml) and in the other almost completely absent (Figure 1). Even though it had been reasonably presumed that they originated from the same neoplastic disease, the different response to chemotherapy of the upper and lower nodules suggested they had a different histological nature. Before any decision was taken relative to chemotherapy we believed it was mandatory to acquire further information by obtaining a tissue diagnosis of the middle lobe nodules. Consequently, wedge resections of these nodules were performed by a three-port thoracoscopy. An intra-operative frozen section revealed no evidence of carcinoma, but several granulomatous structures with multinucleated giant cells suggestive of sarcoidosis. This unexpected result prompted us to carry out a wedge resection of the upper nodule, as in the case of metastasectomy. An intra-operative frozen section showed it to be an adenocarcinoma. This finding excluded a pharyngeal carcinoma metastasis, and established a diagnosis of primitive lung cancer. In accordance with it we went on to perform an upper lobectomy with systematic nodal dissection. The definitive diagnosis was infiltrating primitive pulmonary adenocarcinoma of the right upper lobe, pT1N0M0G2, and multifocal granulomatous disease of the middle lobe suggestive of sarcoidosis.

## Discussion

Over the last few years a number of international scientific societies like the ACCP have developed evidence-based guidelines for the diagnosis and management of lung cancer. They certainly have a great value in assisting physicians throughout the decision-making process and as such are being increasingly used in clinical practice. Abidance to the guidelines means better outcomes for patients, a standardised approach to a disease of worldwide importance and also legal shielding in the case of alleged malpractice. However, the case we report shows there are unusual and complex situations in which the physician's clinical judgment still plays a key role and that must be addressed in a more flexible manner, even by adopting less conventional approaches.

In our patient the clinical and instrumental data - previous history of neoplasia, metabolic hyperactivity of the three pulmonary nodules and positive cytology of the upper nodule - strongly pointed to metastatic pharyngeal carcinoma but intra-operative diagnostics painted a different picture altogether. In fact only one nodule was truly neoplastic and indeed it was not a metastasis but an early-stage primary lung cancer. This information changed the prognosis and the therapeutic strategy. Thus, correct diagnosis and management were possible only thanks to a surgical approach that is commonly inappropriate in such context but was justified by unexpected findings on post-chemotherapy CT and PET imaging.

## Conclusions

In the current era of thoracoscopy, when non-invasive diagnostic tools yield incongruous results surgery can give an important contribution to the diagnosis of lung diseases. The invasiveness of endoscopic surgery is minimal and risks are more than outweighed by benefits, namely saving time and avoiding excessive instrumental exams, misdiagnoses and inappropriate treatments.

## Consent

Written informed consent was obtained from the patient for publication of this case report and accompanying images.

## Competing interests

The authors declare that they have no competing interests.

## Authors' contributions

UC participated in the design of the study and drafted the manuscript, FR participated to the surgical operation and the patient's management, MDS participated in the design of the study, VV participated to the patient's management, MMC participated in the design of the study, AB performed the surgical operation, participated to the patient's management, participated in the design of the study and drafted the manuscript. All the Authors read and approved the final manuscript.
